# Supplementary figures and images for: Introgression between ecologically distinct species following increased salinity in the Colorado Delta- Worldwide implications for impacted estuary diversity
Source: PeerJ. 2017 Dec 12;5:e4056. doi: 10.7717/peerj.4056 (PMC5731342; doi:10.7717/peerj.4056)

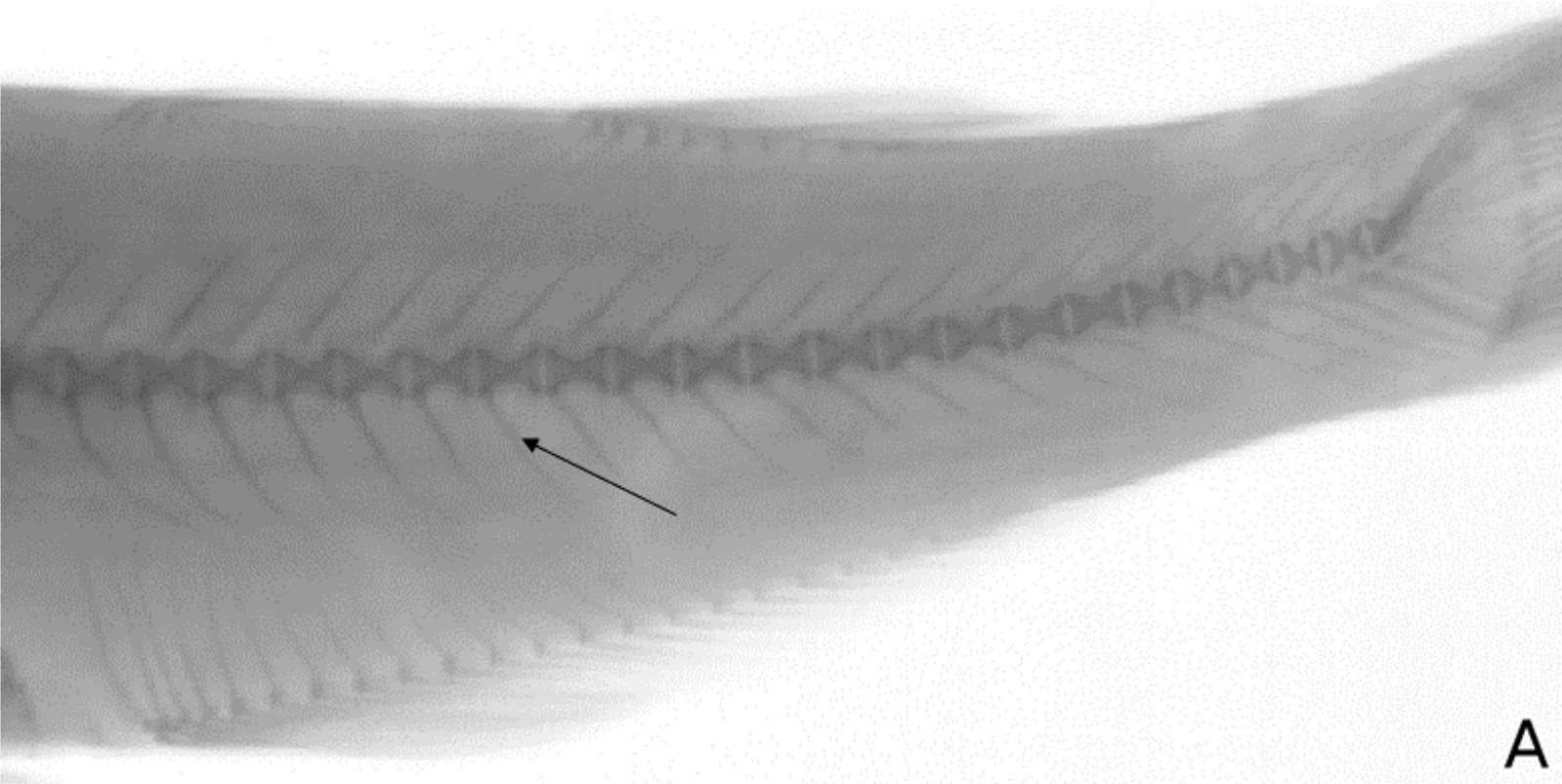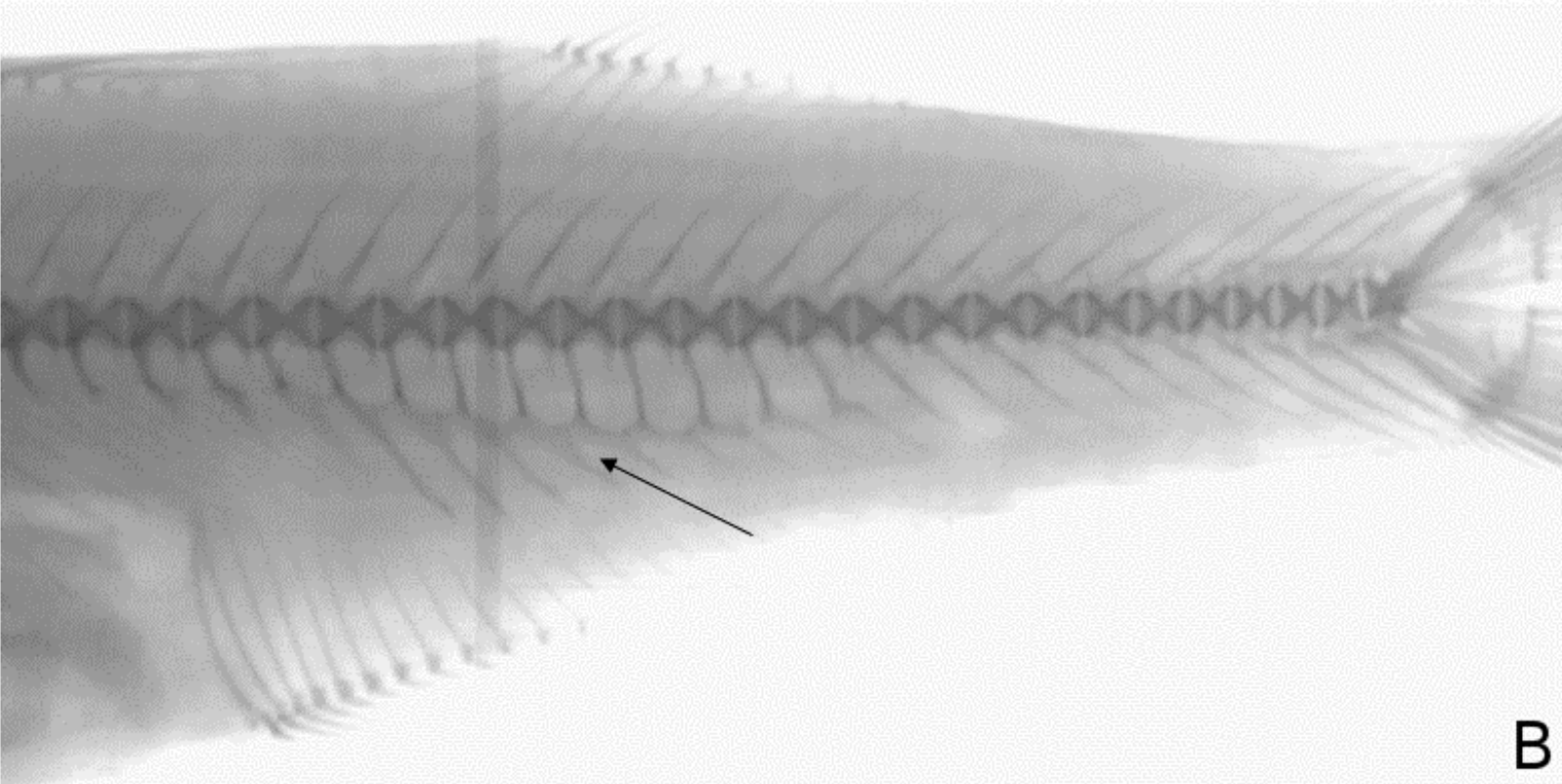

Supplement: Figure S1 — Radiographs of a C. hubbsi specimen (A) and a C. regis specimen (B); arrow in (B) shows the expanded process in the hemal spines of C. regis. [file peerj-05-4056-s001.pdf]

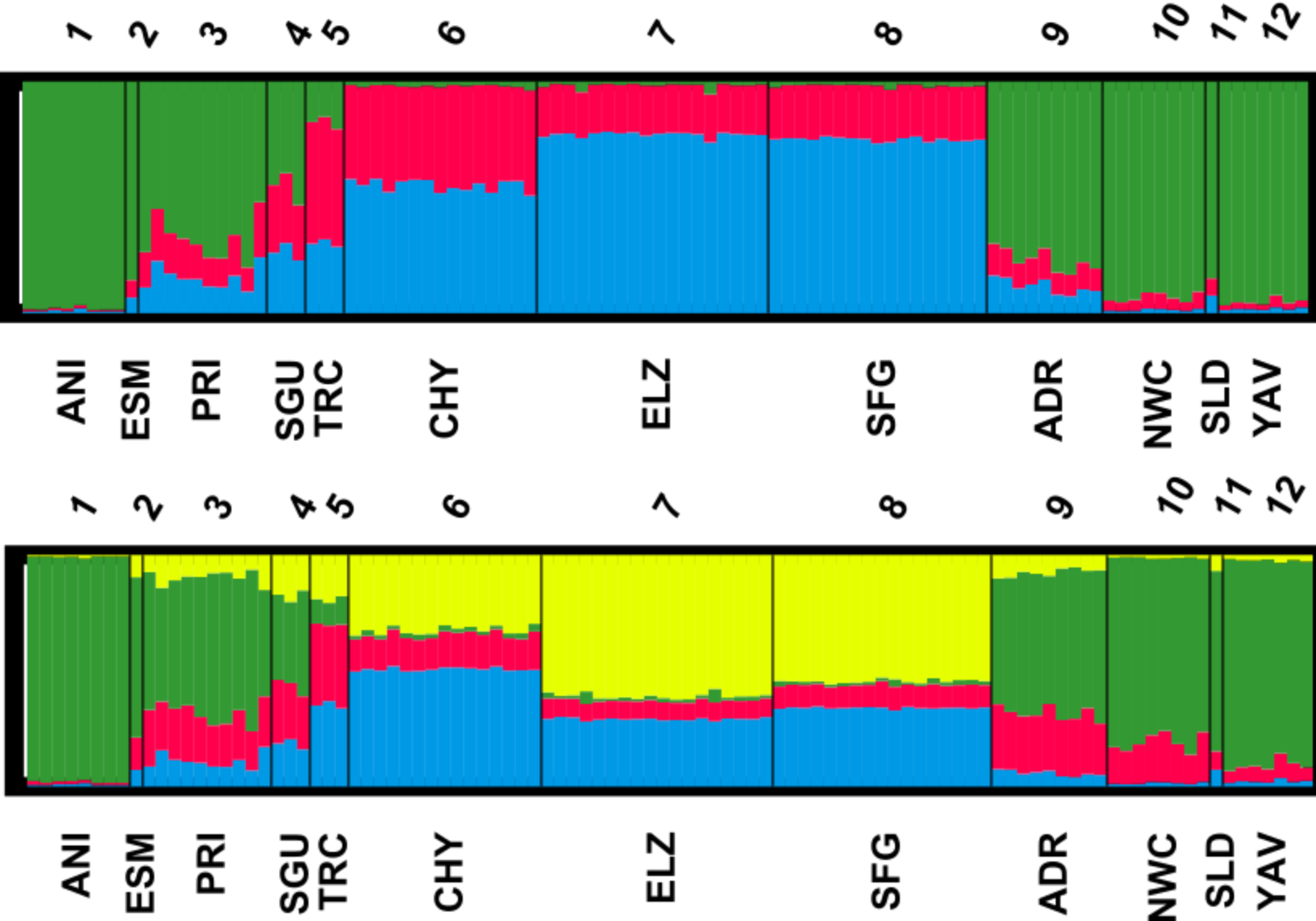

Supplement: Figure S2 — Structure plots for the correlated allele frequency model for K = 3 (top) and K = 4 (bottom). [file peerj-05-4056-s002.pdf]

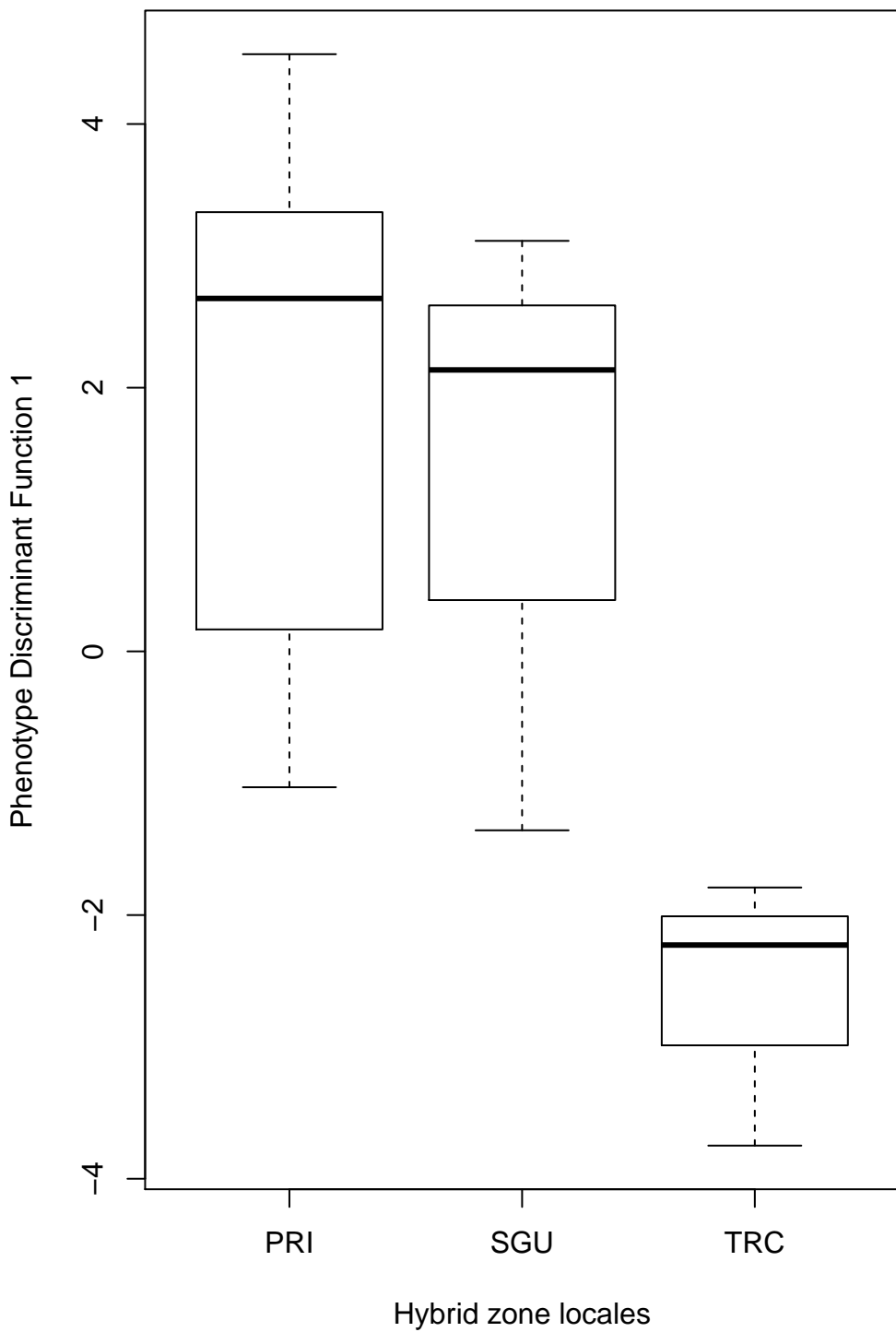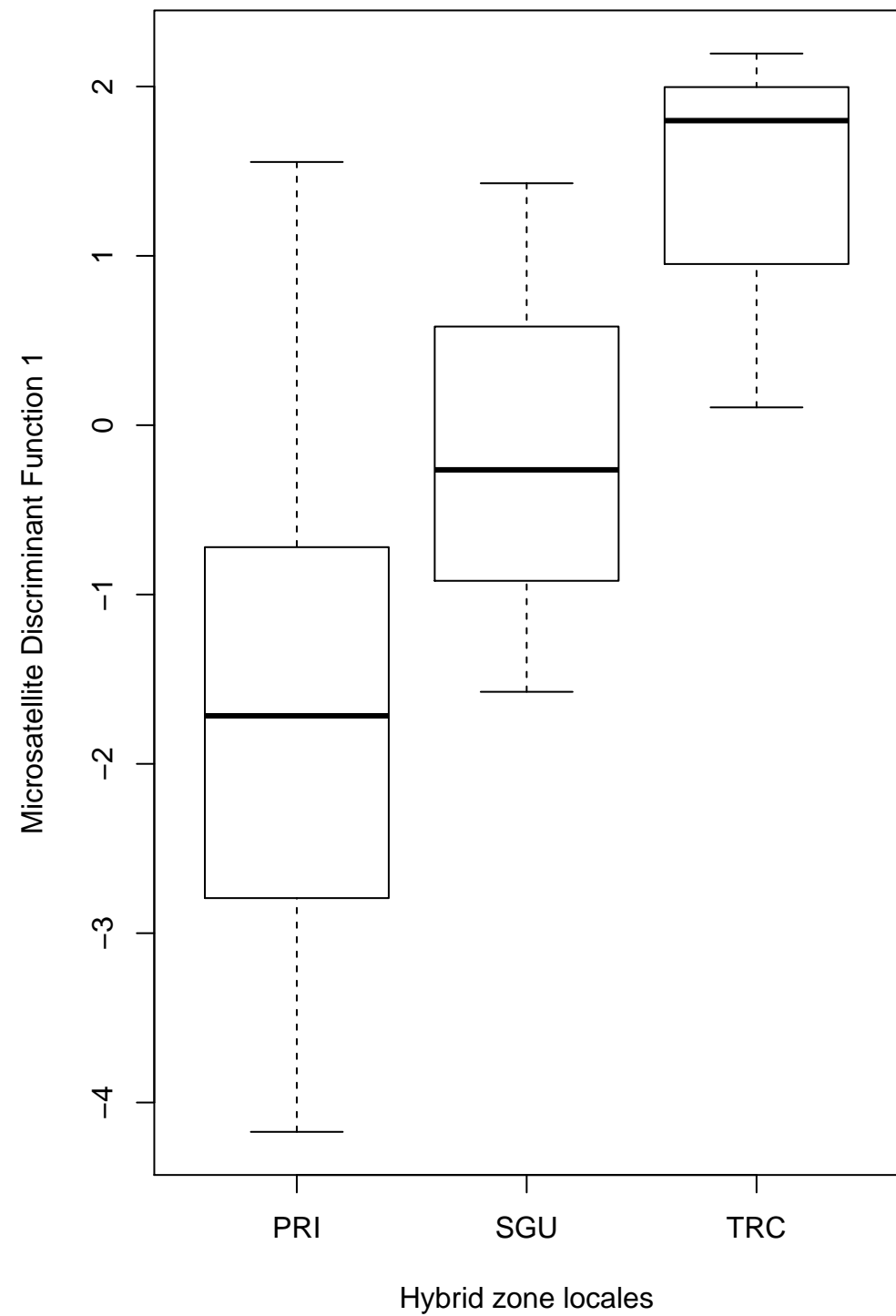

Supplement: Figure S3 [file peerj-05-4056-s003.pdf]
