# Supplementary material for: Introgression between ecologically distinct species following increased salinity in the Colorado Delta- Worldwide implications for impacted estuary diversity
Source: PeerJ. 2017 Dec 12;5:e4056. doi: 10.7717/peerj.4056 (PMC5731342; doi:10.7717/peerj.4056)
Supplement: Table S1 — Measurements of the external morphology of C. regis specimens collected in 1968 at the San Felipe site. SL, standard length; Snout-D1, length from snout to first dorsal fin origin; DLS, dorsolateral scale count. Measurements for specimen 16 (in bold) identify it as C. hubbsi sensu Crabtree (1989). Specimens on loan from the Scripps Institute of Oceanography (SIO) collection. [file peerj-05-4056-s004.docx]

**Supplementary Materials (Tables)**

| Specimen | SL | Snout-D1 to SL ratio | DLS |
| --- | --- | --- | --- |
| SIO 68-46 1 | 117.6 | 0.56 | 49 |
| SIO 68-46 2 | 113.5 | 0.55 | 49 |
| SIO 68-46 3 | 124.3 | 0.53 | 48 |
| SIO 68-46 4 | 138.1 | 0.55 | 51 |
| SIO 68-46 5 | 144.4 | 0.53 | 51 |
| SIO 68-46 6 | 123.8 | 0.54 | 51 |
| SIO 68-46 7 | 126.5 | 0.54 | 50 |
| SIO 68-46 8 | 120.4 | 0.54 | 48 |
| SIO 68-46 9 | 119.8 | 0.58 | 48 |
| SIO 68-46 10 | 115.65 | 0.54 | 50 |
| SIO 68-46 11 | 121.45 | 0.54 | 53 |
| SIO 68-46 12 | 125.9 | 0.54 | 51 |
| SIO 68-46 13 | 116.6 | 0.55 | 50 |
| SIO 68-46 14 | 119.1 | 0.53 | 51 |
| SIO 68-46 15 | 116.4 | 0.55 | 49 |
| SIO 68-46 16 | **126.7** | **0.60** | **44** |
