# Supplementary material for: Introgression between ecologically distinct species following increased salinity in the Colorado Delta- Worldwide implications for impacted estuary diversity
Source: PeerJ. 2017 Dec 12;5:e4056. doi: 10.7717/peerj.4056 (PMC5731342; doi:10.7717/peerj.4056)
Supplement: Table S2 — Frequencies of each microsatellite alleles at each sampling locale. Microsatellite alleles are arranged by size. Number in parentheses under each locale denotes number of individuals analyzed. [file peerj-05-4056-s005.docx]

| Allele | Baja Coast | | Delta Edge | | Colorado River Delta | | | | Sonora Coast | | | |
| --- | --- | --- | --- | --- | --- | --- | --- | --- | --- | --- | --- | --- |
| Odont08 | **ANI**  **(9)** | **ESM**  **(1)** | **PRI**  **(10)** | **SGU**  **(3)** | **TRC**  **(3)** | **CHY**  **(15)** | **ELZ**  **(18)** | **SFG**  **(19)** | **ADR**  **(9)** | **NWC**  **(8)** | **SLD**  **(1)** | **YAV**  **(7)** |
| 146 | - | - | - | - | - | 0.033 | - | - | - | - | - | - |
| 148 | - | - | 0.1 | 0.333 | 0.5 | - | - | - | - | 0.063 | - | - |
| 150 | - | 0.5 | - | - | - | 0.233 | 0.5 | 0.324 | 0.333 | - | - | 0.143 |
| 152 | - | - | - | - | - | 0.133 | 0.056 | 0.029 | - | - | - | - |
| 154 | 0.111 | - | 0.05 | - | 0.333 | 0.367 | 0.028 | 0.029 | - | 0.063 | - | 0.214 |
| 156 | - | - | 0.05 | - | - | - | - | 0.029 | - | - | - | - |
| 158 | 0.167 | 0.5 | 0.25 | - | - | 0.1 | 0.056 | 0.147 | 0.333 | 0.438 | 0.5 | 0.429 |
| 160 | 0.167 | - | 0.2 | - | - | 0.133 | 0.111 | 0.206 | - | - | - | 0.071 |
| 162 | 0.111 | - | 0.1 | 0.333 | 0.167 | - | 0.111 | - | 0.111 | 0.125 | - | - |
| 164 | 0.056 | - | 0.15 | 0.333 | - | - | 0.083 | 0.059 | 0.056 | 0.188 | 0.5 | 0.143 |
| 166 | - | - | 0.1 | - | - | - | 0.028 | 0.089 | 0.056 | 0.125 | - | - |
| 168 | - | - | - | - | - | - | 0.028 | 0.029 | - | - | - | - |
| 170 | 0.056 | - | - | - | - | - | - | - | - | - | - | - |
| Odont09 | **ANI**  **(9)** | **ESM**  **(1)** | **PRI**  **(10)** | **SGU**  **(3)** | **TRC**  **(3)** | **CHY**  **(15)** | **ELZ**  **(18)** | **SFG**  **(19)** | **ADR**  **(9)** | **NWC**  **(8)** | **SLD**  **(1)** | **YAV**  **(7)** |
| 160 | - | - | - | - | - | - | - | 0.029 | - | - | - | - |
| 161 | - | - | - | - | - | - | 0.028 | 0.029 | - | - | - | - |
| 165 | - | - | - | - | - | - | 0.056 | - | - | - | - | - |
| 171 | - | 0.5 | - | 0.167 | 0.167 | - | 0.168 | 0.029 | - | 0.125 | - | 0.071 |
| 173 | 1 | 0.5 | 0.95 | 0.833 | 0.833 | 0.867 | 0.667 | 0.824 | 1 | 0.875 | 1 | 0.929 |
| 175 | - | - | 0.05 | - | - | 0.133 | 0.056 | 0.059 | - | - | - | - |
| 179 | - | - | - | - | - | - | 0.028 | 0.029 | - | - | - | - |
| Odont11 | **ANI**  **(9)** | **ESM**  **(1)** | **PRI**  **(10)** | **SGU**  **(3)** | **TRC**  **(3)** | **CHY**  **(15)** | **ELZ**  **(18)** | **SFG**  **(19)** | **ADR**  **(9)** | **NWC**  **(8)** | **SLD**  **(1)** | **YAV**  **(7)** |
| 154 | - | - | 0.05 | - | 0.167 | 0.033 | 0.028 | 0.059 | 0.056 | - | - | - |
| 156 | 1 | 1 | 0.9 | 0.833 | 0.667 | 0.767 | 0.806 | 0.853 | 0.889 | 1 | 1 | 1 |
| 158 | - | - | 0.05 | 0.167 | 0.167 | 0.2 | 0.167 | 0.089 | 0.056 | - | - | - |
| B18 | **ANI**  **(9)** | **ESM**  **(1)** | **PRI**  **(10)** | **SGU**  **(3)** | **TRC**  **(3)** | **CHY**  **(15)** | **ELZ**  **(18)** | **SFG**  **(19)** | **ADR**  **(9)** | **NWC**  **(8)** | **SLD**  **(1)** | **YAV**  **(7)** |
| 275 | - | - | - | - | - | 0.033 | - | - | 0.056 | 0.063 | - | - |
| 277 | - | - | - | - | - | - | - | 0.088 | 0.056 | - | - | - |
| 279 | - | - | - | - | - | - | 0.083 | 0.059 | - | - | - | - |
| 281 | 0.222 | - | 0.25 | 0.167 | - | 0.1 | 0.056 | 0.059 | 0.222 | - | - | 0.357 |
| 283 | 0.278 | 0.5 | 0.2 | - | 0.333 | 0.033 | 0.028 | 0.059 | 0.056 | 0.063 | - | 0.071 |
| 285 | - | - | 0.05 | - | 0.167 | 0.1 | - | 0.059 | 0.167 | 0.25 | - | 0.071 |
| 287 | - | - | 0.05 | 0.167 | - | 0.033 | 0.139 | 0.118 | - | 0.125 | 0.5 | 0.286 |
| 289 | 0.5 | 0.5 | 0.1 | 0.167 | - | 0.067 | 0.194 | 0.059 | 0.167 | 0.25 | - | 0.143 |
| 291 | - | - | 0.05 | - | 0.167 | 0.067 | 0.083 | 0.176 | - | - | 0.5 | - |
| 293 | - | - | 0.1 | - | - | 0.1 | 0.028 | 0.176 | 0.056 | 0.125 | - | - |
| 295 | - | - | 0.1 | 0.167 | - | 0.1 | 0.028 | 0.029 | 0.167 | 0.125 | - | - |
| 297 | - | - | - | - | - | 0.033 | 0.056 | - | - | - | - | 0.071 |
| 299 | - | - | 0.1 | 0.167 | - | 0.067 | 0.028 | - | - | - | - | - |
| 302 | - | - | - | - | - | - | 0.028 | 0.029 | - | - | - | - |
| 304 | - | - | - | - | - | 0.1 | 0.056 | - | - | - | - | - |
| 306 | - | - | - | 0.167 | 0.167 | 0.033 | 0.056 | 0.059 | - | - | - | - |
| 310 | - | - | - | - | - | 0.033 | - | - | - | - | - | - |
| 313 | - | - | - | - | - | - | - | - | 0.056 | - | - | - |
| 314 | - | - | - | - | - | - | - | 0.029 | - | - | - | - |
| 316 | - | - | - | - | 0.167 | 0.067 | - | - | - | - | - | - |
| 318 | - | - | - | - | - | 0.033 | 0.056 | - | - | - | - | - |
| 320 | - | - | - | - | - | - | 0.028 | - | - | - | - | - |
| 331 | - | - | - | - | - | - | 0.028 | - | - | - | - | - |
| 333 | - | - | - | - | - | - | 0.028 | - | - | - | - | - |
| B19 | **ANI**  **(9)** | **ESM**  **(1)** | **PRI**  **(10)** | **SGU**  **(3)** | **TRC**  **(3)** | **CHY**  **(15)** | **ELZ**  **(18)** | **SFG**  **(19)** | **ADR**  **(9)** | **NWC**  **(8)** | **SLD**  **(1)** | **YAV**  **(7)** |
| 224 | - | - | - | 0.333 | - | 0.2 | 0.222 | 0.235 | -- | - | - | - |
| 226 | - | - | - | - | - | 0.033 | 0.028 | 0.029 | 0.056 | - | - | - |
| 228 | - | - | 0.3 | 0.167 | 0.667 | 0.267 | 0.222 | 0.324 | 0.167 | 0.063 | - | - |
| 230 | - | - | - | 0.167 | 0.167 | 0.133 | 0.25 | 0.147 | - | - | - | - |
| 232 | - | 0.5 | 0.3 | - | 0.167 | 0.3 | 0.222 | 0.235 | 0.333 | 0.125 | - | 0.143 |
| 234 | 0.889 | 0.5 | 0.4 | 0.167 | - | 0.067 | 0.056 | 0.029 | 0.278 | 0.688 | 1 | 0.571 |
| 236 | - | - | - | 0.167 | - | - | - | - | - | - | - | 0.143 |
| 238 | - | - | - | - | - | - | - | - | 0.056 | 0.063 | - | - |
| 240 | - | - | - | - | - | - | - | - | - | 0.063 | - | 0.143 |
| Table continued on next page. | | | | | | | | | | | | |
|  |  |  |  |  |  |  |  |  |  |  |  |  |
| Table continued from previous page. | | | | | | | | | | | | |
| B39 | **ANI**  **(9)** | **ESM**  **(1)** | **PRI**  **(10)** | **SGU**  **(3)** | **TRC**  **(3)** | **CHY**  **(15)** | **ELZ**  **(18)** | **SFG**  **(19)** | **ADR**  **(9)** | **NWC**  **(8)** | **SLD**  **(1)** | **YAV**  **(7)** |
| 252 | 1 | 1 | 0.75 | 0.667 | 0.333 | 0.033 | 0.028 | 0.029 | 1 | 1 | 1 | 1 |
| 254 | - | - | 0.1 | 0.333 | 0.667 | 0.733 | 0.528 | 0.618 | - | - | - | - |
| 256 | - | - | - | - | - | 0.133 | 0.139 | 0.176 | - | - | - | - |
| 258 | - | - | 0.05 | - | - | - | 0.139 | 0.29 | - | - | - | - |
| 262 | - | - | - | - | - | 0.033 | - | - | - | - | - | - |
| 264 | - | - | 0.05 | - | - | - | 0.0833 | 0.059 | - | - | - | - |
| 266 | - | - | - | - | - | - | 0.056 | 0.059 | - | - | - | - |
| 268 | - | - | 0.05 | - | - | 0.033 | 0.028 | - | - | - | - | - |
| 271 | - | - | - | - | - | 0.033 | - | 0.029 | - | - | - | - |
